# *Euphorbia hirta* nanoextract as a piezoelectric ultrasonic scaler coolant in gingivitis treatment in a Wistar rat model

**DOI:** 10.1016/j.jtumed.2023.09.004

**Published:** 2023-10-06

**Authors:** Archadian Nuryanti, Andari Sarasati, Latifah Ulfahastika, Maria Ditya Wartadiani, Muhammad Hidayat Syahruddin, Rachma Nissa Sitho Qurota A'yun

**Affiliations:** a Dental Biomedical Sciences Department, Faculty of Dentistry, Universitas Gadjah Mada, Yogyakarta, Indonesia; b Doctoral Study Program, Faculty of Dentistry, Universitas Gadjah Mada, Yogyakarta, Indonesia; c Dentistry Study Program, Faculty of Dentistry, Universitas Gadjah Mada, Yogyakarta, Indonesia

**Keywords:** الفربيون هيرتا, عامل التبريد, التهاب اللثة, تكوين الأوعية الدموية, العدلات, Angiogenesis, Coolant, *Euphorbia hirta*, Gingivitis, Neutrophil

## Abstract

**Objectives:**

This research was aimed at investigating the effects of various concentrations of *Euphorbia hirta* nanoextract as a piezoelectric scaler coolant on gingivitis healing in a Wistar rat model.

**Methods:**

A piezoelectric ultrasonic scaler coolant was made from *E. hirta* nanoextract through ionic gelation. Experiments were conducted in 45 adult male Wistar rats divided into three groups treated with *E. hirta* nanoextract coolant (25%, 30%, and 35% concentrations), and negative and positive control groups. A silk ligature was used to trap debris and induce gingivitis in the maxillary incisors of the rats. Scaling was conducted with a piezoelectric ultrasonic scaler after the respective treatment for each group. Data were collected on days 3, 5, 7, 14, and 21 after treatment. Observations were collected with an Optilab® camera at 400× magnification. Angiogenesis and neutrophil data were analyzed with two-way analysis of variance (ANOVA) and post hoc Duncan tests at a 95% significance level.

**Results:**

Use of *E. hirta* nanoextract as a piezoelectric ultrasonic coolant accelerated gingivitis healing in Wistar rats, particularly at a 25% concentration. Two-way ANOVA indicated a significant difference in angiogenesis and neutrophil counts between the control group and each treatment group (p < 0.05). Duncan's post-hoc test showed significant differences in mean neutrophil numbers and angiogenesis among groups on days 3, 5, 7, 14, and 21. The group treated with 25% nanoextract concentration showed no significant differences with respect to the positive control group.

**Conclusions:**

Use of *E. hirta* nanoextract as a piezoelectric ultrasonic coolant had good therapeutic results in promoting gingivitis healing. *E. hirta* nanoextract may potentially resolve inflammation in gingivitis by modulating neutrophils and angiogenesis.

## الملخص

**أهداف البحث:**

يهدف هذا البحث إلى دراسة تأثير تراكيز مختلفة من مستخلص الفربيون هيرتا النانوي كمبرد قشارة كهرضغطية على شفاء التهاب اللثة في فئران ويستار.

**طريقة البحث:**

تم تحضير الأقراص ثنائية الطبقة عن طريق تحسين اللب والغشاء شبه المنفذ. تم تحليل التأثير الإضافي لوقت المعالجة مع حجم وعدد الفتحة على ملف الذوبان للأقراص المعدة. تم استخدام درجتين مختلفتين من أكسيد البولي إيثلين في الطبقة الأساسية وطبقة الدفع كمشكلين للمسام.

**النتائج:**

عامل تبريد قشارة بالموجات فوق الصوتية الكهرضغطية مصنوع من مستخلص الفربيون هيرتا النانوي بطريقة التهلم الأيونية. تم إجراء ذلك على 45 من ذكور فئران ويستار البالغة. تم تقسيم الحيوانات إلى خمس مجموعات؛ مجموعة معالجة عامل التبريد بمستخلص الفربيون هيرتا النانوي (تركيزات 25% و30% و35% من الفربيون هيرتا)، ومجموعات السيطرة السلبية، ومجموعات المراقبة الإيجابية. تم استخدام رباط حريري لاحتجاز الحطام والحث على التهاب اللثة في القواطع العلوية للفئران. تم إجراء القياس باستخدام قشارة بالموجات فوق الصوتية الكهرضغطية بعد العلاج المناسب لكل مجموعة. تم جمع البيانات في الأيام 3 و5 و7 و14 و21 بعد العلاج. تم إجراء المراقبة باستخدام أوبتيلاب بقدرة تكبير 400اكس. تم تحليل بيانات تكوين الأوعية الدموية والعدلات من خلال تحليل التباين ثنائي الاتجاه، واختبارات دنكان اللاحقة عند مستوى أهمية 95٪.

**الاستنتاجات:**

لقد وجدنا أن مستخلص الفربيون هيرتا النانوي كعامل تبريد بالموجات فوق الصوتية الكهرضغطية يمكن أن يعزز تسريع شفاء التهاب اللثة في فئران ويستار، خاصة عند تركيز 25٪. أظهر تحليل أنوفا ثنائي الاتجاه اختلافا كبيرا في تكوين الأوعية الدموية والعدلات بين المجموعة الضابطة وكل مجموعة علاجية وأظهرت تجربة دنكان اللاحقة اختلافات كبيرة في متوسط عدد العدلات وتولد الأوعية بين المجموعات في الأيام 3،5،7،14، و21. لم تظهر مجموعة العلاج بتركيز مستخلص النانو بنسبة 25% أي اختلاف كبير عن مجموعة التحكم الإيجابية. قدم مستخلص الفربيون هيرتا النانوي كعامل تبريد بالموجات فوق الصوتية الكهرضغطية نتائج علاجية جيدة في تعزيز تأثير الشفاء من التهاب اللثة. من المحتمل أن يعمل مستخلص الفربيون هيرتا النانوي على حل الالتهاب في التهاب اللثة، من خلال التأثير على العدلات وتولد الأوعية.

## Introduction

Gingivitis is a pathological change associated with the presence of microorganisms that adhere to the teeth and accumulate in the gingival sulcus area. Products of pathogenic gingivitis-inducing microorganisms, such as collagenase, hyaluronidase, chondroitin sulfatase, and endotoxins, and substantial amounts of glycolic acid, damage epithelial cells and connective tissue.^1^ These bacterial products activate responses in the body leading to inflammation and tissue destruction.^3^ The symptoms of gingivitis include redness and swelling of the gingiva (bulbous-shaped interdental papillae) and bleeding upon probing.^1^ This condition is progressive, destructive, and reversible.^1^^,^^2^ The symptoms of gingivitis also include halitosis. Sustained gingivitis can develop into periodontitis with signs of attachment loss, periodontal pockets, gingival recession, and tooth mobility.^1^

The prevalence of gingivitis in Indonesia, according to Basic Health Research Indonesia, is 74.1%; thus, gingivitis is among the most common oral health problems in this country.^4^ Scaling and polishing remove deposits (calculus, stain, and debris) on the tooth surface that contain bacterial plaque.^5^ Scaling can remove subgingival deposits, thus inducing gingival healing, including complex cellular, chemical, and vascular tissue processes.^5^^,^^6^ Scaling and root planning are important to eliminate the causative factors of periodontal disease during periodontal therapy that include non-surgical, surgical, and maintenance phases.^7^ However, scaling and root planning cannot fully eliminate certain pathogens, such as *Aggregatibacter actinomycetemcomitans*,^8^ a pathogenic bacterium inducing infections such as gingivitis and periodontitis.^1^ In cases of deep periodontal pockets, additional treatment may include antimicrobial agents, but the effectiveness of antimicrobial treatment and the risk of antibiotic resistance must be considered. Therefore, alternative treatments are needed to maintain a balanced oral microflora and achieve successful periodontal therapy.^8^

*Euphorbia hirta* is a wild plant with bioactive compounds, such as flavonoids, tannins, and saponins. This plant is widely available and has not been optimally used in periodontal therapy.^9^ Phytochemical tests have shown that *E. hirta* contains 206.170 ± 1.950 mg GAE/g total phenolic compounds and 37.970 ± 0.003 mg CE/g flavonoids.^10^ The phenol fraction in *E. hirta* has antifungal and antioxidant effects.^9^^,^^10^ Flavonoids play roles in wound healing by suppressing fibroblast growth factor (FGF)-induced angiogenesis.^10^^,^^11^ These bioactive compounds are a potential natural source of anti-inflammatory agents for treating various inflammatory diseases by accelerating angiogenesis. *E. hirta* is rarely used in periodontal therapy, despite its potential to provide a potential alternative to enhance periodontal therapy efficacy.

Saponins are part of the plant defense system against pathogenic microorganisms. Terpenoids or steroid glycosides conjugate with one or more sugar chains; the glycosidic bond in its structure has biological effects, including antibacterial activity, angiogenesis stimulation, type I collagen generation, and tissue epithelialization. Tannins also have been found to limit secondary infections.^12^^,^^13^ Moreover, *E. hirta* elicits concentration-dependent increases in anti-inflammatory effects by decreasing inflammatory mediators and cells.^14^

Nanoparticle technology can be used to increase drug bioavailability and create controlled drug delivery systems.^15^ Nanoparticle-sized drug formulations have been shown to influence the effectiveness of angiogenesis, even in cases of treatment-resistant cancer.^16^ Nanoparticles, owing to their small size, can interact with various cells and biomolecules, thereby enhancing the delivery and clinical effects of therapeutic agents. Chitosan, a polymeric product of the synthetically produced chitin shells of marine animals, is an example of an encapsulant or coating agent. This compound is biodegradable, biocompatible, food-safe, and easily prepared, and additionally has low toxicity.^17^ Chitosan also has antibacterial effects, particularly against gram-positive bacteria.^18^^,^^19^

The application of *E. hirta* in coolant water has not previously been studied, and its potential remains to be demonstrated. Thus, this study was aimed at exploring the potential of *E. hirta* as a piezoelectric ultrasonic coolant affecting neutrophils and angiogenesis in an animal model.

## Materials and Methods

### Preparation of *E. hirta* extract

*E. hirta* identification was conducted in the Plant Laboratory, Department of Biology, Universitas Gadjah Mada, Special Region of Yogyakarta, Indonesia. *E. hirta* was cultivated in Sleman, Yogyakarta, Indonesia. *E. hirta* extract was obtained with the maceration method. The fifth leaves from the roots were removed, and 1.59 kg was weighed. After being washed, the leaves were dried for 24 h at 50 °C. Dried leaves were crushed to obtain powder (250 g). Maceration was performed by soaking the *E. hirta* powder in 70% ethanol in a 1:10 ratio. The solvent was removed with a rotary evaporator at 40 °C and 90 rpm. The resultant 75 g of extract was stored in a sterile container at −10 °C until use.

### Preparation of *E. hirta* nanoextract

*E. hirta* nanoextract preparation was performed with the chitosan encapsulation ionic gelation method. Two grams of *E. hirta* extract was dissolved in 0.2% chitosan solution with an Ultraturax homogenizer for 30 min with gradual addition of 40 mL of 0.1% NaTPP solution. Homogenates were centrifuged to obtain *E. hirta* nanoextract. The obtained *E. hirta* nanoextract underwent size characterization at the Nanoparticle Technology Laboratory, Universitas Islam Indonesia, Yogyakarta, Republic of Indonesia, performed with a HORIBA Scientific SZ-100 instrument. The *E. hirta* nanoextract was then dissolved in 0.1% ethanol to concentrations of 25%, 30%, and 35%.

### Sample size determination

The necessary sample size was determined with the following calculation.^20^

$$\text{Minimum}\;n=\frac{10}{\left(k\right)\left(r\right)}+1$$

$$=\frac{10}{\left(5\right)\left(3\right)}+1$$

=1.67∼2

$$\text{Maximum}\;n=\frac{20}{\left(k\right)\left(r\right)}+1$$

$$=\frac{20}{\left(5\right)\left(3\right)}+1$$

=2.33–3

Minimumn=(minimumn)(k)(r)

=(2)(5)(3)

=30

Maximumn=(maximumn)(k)(r)

=(3)(5)(3)

=45

N = total number of rats
n = number of rats per group
k = number of groups
r = number of measurements

### Experimental model and housing

Forty-five male adult Wistar rats, purchased from a local supplier, 2–3 months of age and weighing 200–250 g, were randomly selected. The rats were fed AD2 pellets and given reverse osmosis purified water. They were divided into a negative control group (water coolant); a positive control group (2% iodine glycerin coolant); and three groups treated with nanoextract concentrations of 25%, 30%, and 35%. Each group was randomly divided into five observation-period subgroups, (3, 5, 7, 14, and 21 post-treatment days) with three animals each. The Wistar rats were housed under a constant temperature of 24 °C with good ventilation. The rats were monitored by a veterinarian throughout the experiment and were in good health. Intramuscular doses 10 mg/kg of ketamine hydrochloride and xylazine 2 mg/kg were administered to the thigh muscles of each animal.

### Treatment procedure

After anesthesia, a silk ligature (reverse cutting 4.0 3/8) was placed in the central incisor area to promote debris accumulation inducing gingivitis. The silk ligature was left in place for 7 days until characteristics of gingivitis (redness, edema, and bleeding on probing) appeared. Rats in the negative control group (scaling with water coolant water only), treatment groups (25%, 30%, or 35% nanoextract coolant), and positive control group (scaling with 2% iodine glycerin coolant) were anesthetized to remove the silk ligature, and scaling was performed. At the specified observation times, the animals were euthanized, and maxillary and mandibular histological samples were obtained, particularly from the incisor area. The tissue in the central incisor area, including the central incisor teeth, gingiva, and periodontal bone, was collected. The samples were fixed in phosphate-buffered formalin.

### Histological examination and image analysis

Maxillary and mandibular periodontal tissue were decalcified with Von Ebner liquid for 24 h. Coarse slicing was performed with a scalpel (thickness 0.5 mm), and dehydration was subsequently performed with a graded alcohol series (70%, 80%, 90%, and 100%) for 60 min. The tissue was cleaned with xylol. Samples were placed under vacuum for 30 min to remove air from the tissue, then embedded in a paraffin block (created at 60 °C). The paraffin block was sliced with a microtome to 4 μm. Hematoxylin–eosin staining was performed to identify neutrophils and angiogenesis. Angiogenesis and neutrophil numbers were calculated with a light microscope and Optilab® digital camera. Neutrophil numbers and angiogenesis were calculated on days 3, 5, 7, 14, and 21 in three visual fields in the junctional area of the epithelium and gingival pocket, where neutrophil cell infiltration and angiogenesis processes occurred. The gingival histological specimens were observed at a magnification of 400× in three fields of view by three different observers.

### Statistical analysis

Data were statistically analyzed in the SPSS 22.0 software package for Windows. Shapiro–Wilk test was used to determine the normality, and Levene's test was used to determine the homogeneity of the data. The data are presented as mean and standard deviation for each measurement in the control and treatment groups. Furthermore, the data were analyzed with two-way ANOVA with a significance threshold of p < 0.05, followed by a post-hoc Duncan test, to examine differences among groups.

## Results

### *E. hirta* nanoparticle synthesis and characterization

Particle size analysis indicated an *E. hirta* nanoextract particle size of 485 nm and a zeta potential of 43.6 mV. Nanoparticles have diameters of 10–1000 nm in colloids or solids.^21^ A zeta potential higher than ±30 mV facilitates the stability of nanoemulsion droplets in a system, thus preventing flocculation.^22^

### Histological appearance of neutrophils and angiogenesis

**Figure 1:**
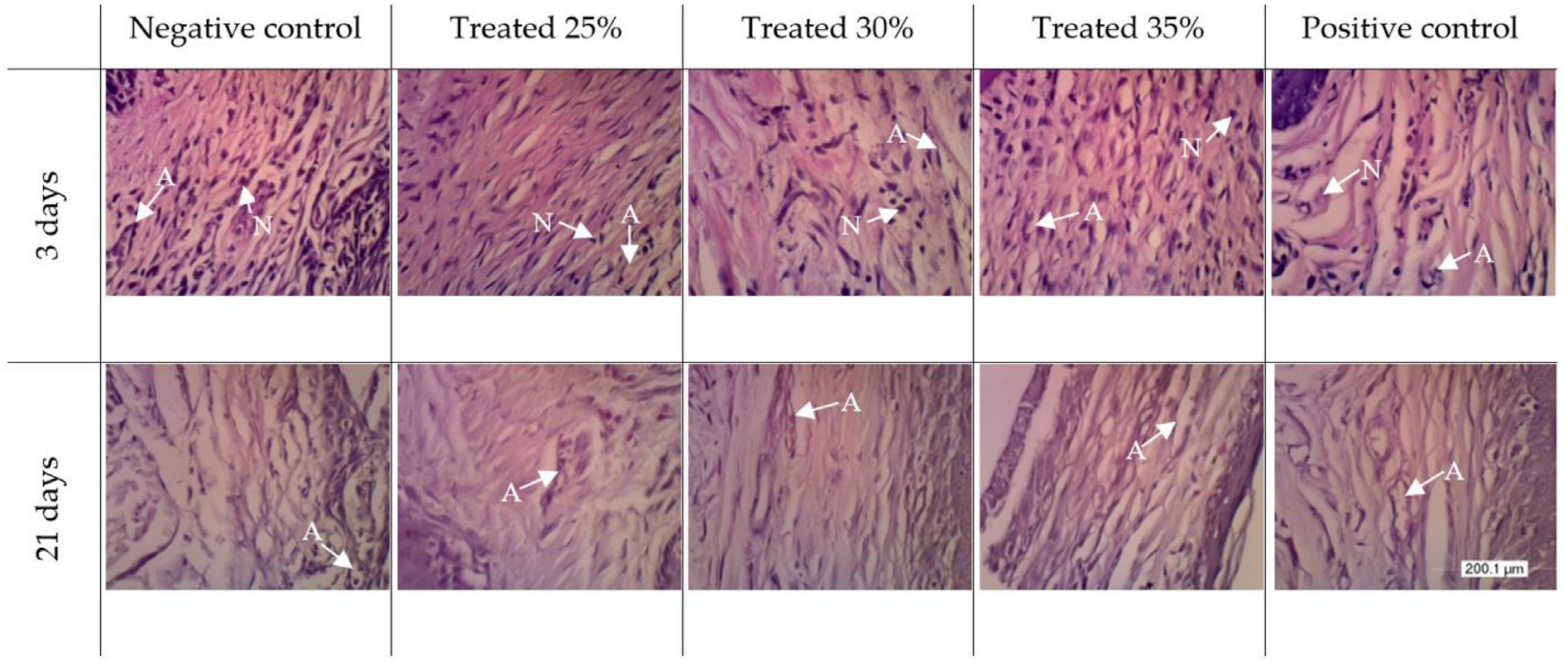
Histopathological appearance (400×) of gingival tissue on days 3 and 21 after treatment in the (1) negative, (2) 25%, (3) 30%, (4) 35%, and (5) positive groups. An arrow points to the events of gingival healing. A: angiogenesis; N: neutrophils.

**Figure 2:**
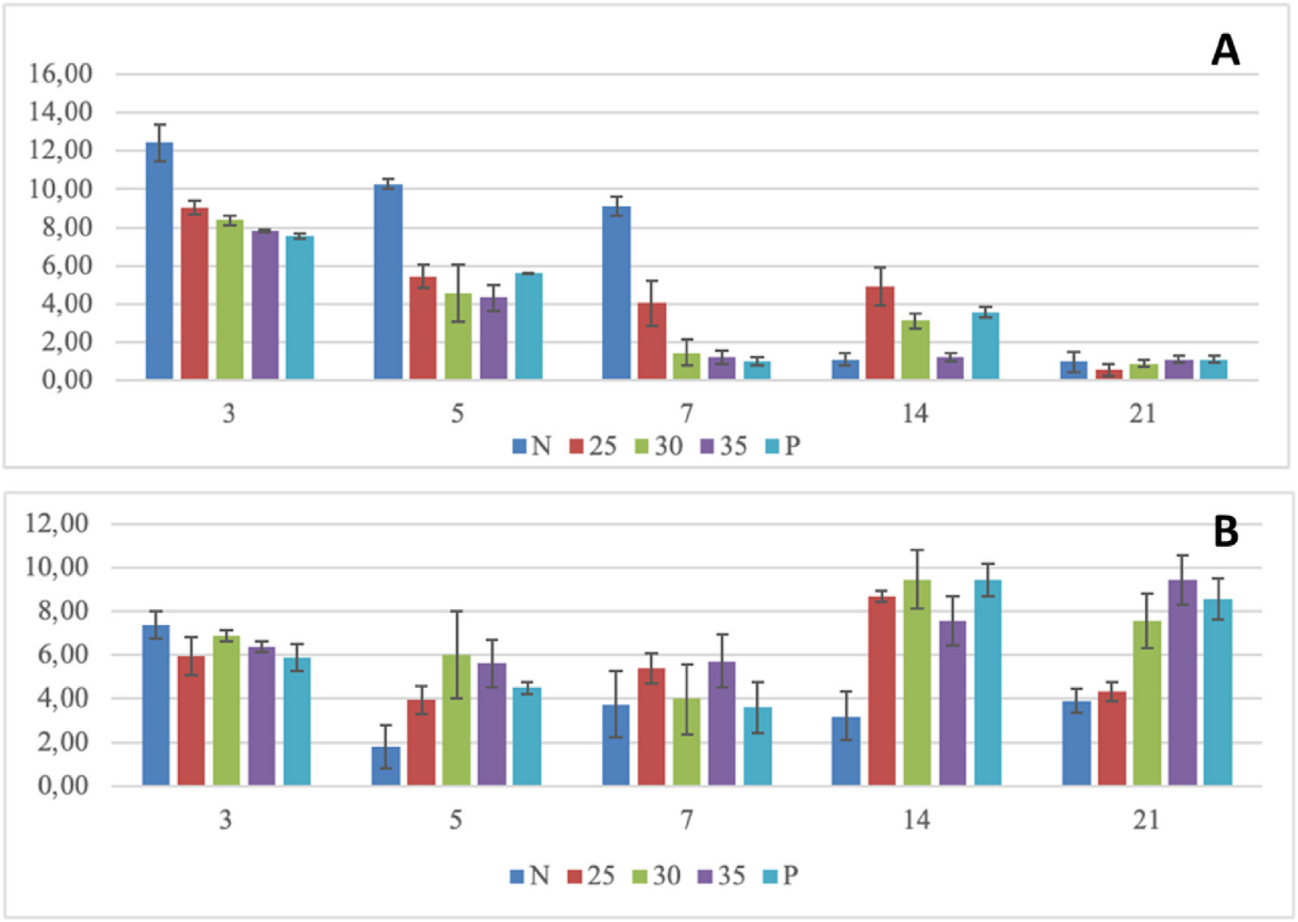
Mean (A) neutrophil number and (B) angiogenesis on days 3, 5, 7, 14, and 21 in the negative group; groups treated with 25%, 30%, or 35% *E. hirta* nanoextract coolant water; and the positive group.

Hematoxylin–eosin staining showed that the neutrophils had dark-purple staining, were oval in shape, and contained multiple nuclei (Figure 1). Angiogenesis is the process of formation of new blood vessels. Blood vessels appear as cavities filled or without red erythrocytes surrounded by endothelial cells.^23^ Mean ± standard deviation neutrophil counts are shown in Figure 2A, and angiogenesis is shown in Figure 2B.

### Data analysis

#### Numbers of neutrophils

The highest mean number of neutrophils was found on day 3, and subsequently decreased on days 5, 7, 14, and 21 in all groups. The Shapiro–Wilk test indicated that the data were normally distributed with a p-value >0.05. Levene's test showed that the data were homogeneous with a p-value >0.05. Two-way ANOVA revealed significant differences among all research groups (p = 0.000) (Table 1). Duncan's post hoc test (Table 2) showed no differences in neutrophil quantity and angiogenesis among groups on day 3. Significant differences were observed between the control groups and treatment groups (Table 2, p < 0.05), and the mean number of neutrophils decreased by day 5. Significant mean differences were found between the negative control and 25% treatment groups, but no difference was found between the negative control group and the 30% and 35% treatment groups (Table 2, p > 0.05). The 25% *E. hirta* nanoextract groups showed decreases in neutrophils by days 7, 14, and 21.

**Table 1:** Two-way ANOVA for neutrophil data.

Source	df	Mean square	F	Sig.
Treatment	4	59.89	117.57	0.000∗
Day	4	121.50	238.51	0.000∗
Treatment × day	16	2.54	5.00	0.000∗

(∗): significance (p < 0.05).

**Table 2:** Duncan's post-hoc test for neutrophil data.

Day	Negative control	25%	30%	35%	Positive control
3	9.05^g^	8.38^fg^	7.83^f^	7.55^f^	7.55^g^
5	9.11^g^	5.44^cde^	4.55^cd^	4.33^c^	5.61^e^
7	10.27^h^	4.05^c^	1.44^a^	1.22^a^	1.00^a^
14	1.00^a^	2.00^a^	3.00^b^	4.00^c^	5.00^cde^
21	1.00^a^	2.00^a^	3.00^b^	4.00^c^	5.00^cde^

Note: Numbers followed by different letters indicate significant differences (p < 0.05, e.g., p = 0.00).

As shown in Table 1 and Figure 2, the effects of *E. hirta* nanoextract were observed at the indicated healing times. Inflammatory cells, particularly neutrophils, were recruited and appeared in the first 3 days, and continued to be recruited until day 7.

#### Angiogenesis

The angiogenesis data showed a normal distribution (p > 0.05) and were homogeneous (p > 0.05). Two-way ANOVA indicated that the mean angiogenesis in all groups decreased by day 3 and subsequently increased by day 5 in the 30% and 35% treatment groups (Table 3). The mean angiogenesis of all groups increased by day 7. The highest angiogenesis occurred on day 14 and subsequently decreased by day 21. Duncan's post hoc test indicated no significant differences in mean angiogenesis on day 3 among groups (Table 4). On day 5, a significant difference in angiogenesis between the control and treatment groups (30% and 35%) was observed. Treatment with 25% *E. hirta* nanoextract had the same effects as those observed in the positive control group starting on day 5 and continuing through day 14. This result suggested that 25% *E. hirta* nanoextract had superior effects against gingivitis. As shown in Table 2 and Figure 2, the effects of *E. hirta* nanoextract on gingivitis in Wistar rats were demonstrated to promote angiogenesis and healing of the gingiva.

**Table 3:** Two-way ANOVA for angiogenesis data.

Source	df	Mean square	F	Sig.
Treatment	4	3.835	3.223	0.000∗
Day	4	21.297	17.895	0.000∗
Treatment × day	16	4.199	3.528	0.000∗

(∗): significance (p < 0.05).

**Table 4:** Duncan's post-hoc test for angiogenesis data.

Day	Negative control	25%	30%	35%	Positive control
3	7.38^defgh^	5.94^bcdefg^	6.89^cdefg^	6.38^cdefg^	5.89^bcdefg^
5	1.83^a^	3.94^abc^	6.00^bcdefg^	5.61^bcde^	4.50^abcd^
7	3.77^abc^	5.38^bcde^	4.00^abc^	5.72^bcdef^	3.61^abc^
14	3.22^ab^	9.44^h^	8.66 g^h^	7.55^efgh^	9.44^h^
21	3.88^abc^	4.33^abc^	7.55^efgh^	9.44^h^	8.55^fgh^

Note: Numbers followed by different letters indicate significant differences (p < 0.05, e.g., p = 0.00).

## Discussion

Use of *E. hirta* nanoextract as a piezoelectric ultrasonic scaler coolant was found to affect neutrophil number and angiogenesis in the gingiva in rats with gingivitis. The mean number of neutrophils was lower in the gingiva in rats with gingivitis treated with *E. hirta* nanoextract than with 2% iodine glycerin and distilled water (Figure 2A). Higher mean angiogenesis was observed in the gingiva in rats in the treatment than the control groups. In addition, two way-ANOVA demonstrated significant differences in neutrophil numbers and angiogenesis between the control and treatment groups. Significant differences were indicated by Duncan's post hoc test among the gingivitis groups given distilled water, *E. hirta* extract, and 2% iodine glycerin (Table 2, Table 4).

The increase in mean neutrophil number on day 3 indicated an initial inflammatory response in the form of neutrophil migration to the area of infection in the first 24 h. The accumulated plaques were composed of bacteria, and their metabolic products induced a gingival inflammatory process characterized by erythema, edema, and bleeding upon probing. The neutrophil number peaked in the initial 48−72 h. In infection areas, neutrophils enable the phagocytosis of bacteria and debris. Neutrophils undergo apoptosis, decrease in number, and are replaced by monocytes.^21^^,^^22^^,^^24^ The migrated monocytes transform into macrophages that clean up matrix debris and tissue, including neutrophils that have undergone apoptosis.^25^ Neutrophil clearance by monocytes and a decreased inflammatory cell count indicate healing of gingivitis.

Gingivitis has three development stages: initial lesions, early lesions, and established lesions. The initial lesions occur 2–4 days after plaque accumulation, wherein the histological changes begin with transient vasoconstriction followed by dilatation of capillary vasculature, arteries, and venules in the dentogingival plexus. These vascularization changes result in the exudation of fluid and protein into tissue; subsequently hazardous substances from bacterial products infiltrate the tissue. The next stage is early lesions, which occur 4−7 days after plaque accumulation. This stage is associated with capillary proliferation and the formation of capillary circles between rete ridges, thus resulting in bleeding upon probing. Gingiva clinical signs begin to appear at this stage. The last stage is established lesions, which occur 2−3 weeks after plaque accumulation, as indicated by gingival erythema that becomes bluish; bleeds upon probing; and shows changes in consistency and texture, and increased gingiva sulcus depth. This stage can remain stable for several years but can also develop into destructive lesions, which cause deeper periodontal tissue damage or periodontitis.^1^

The negative control group given distilled water had a significantly higher number of neutrophils than the other groups of rats (p < 0.05), thus suggesting that distilled water does not have antibacterial or anti-inflammatory effects. Significant differences in the mean numbers of neutrophils among groups suggested that *E. hirta* nanoextract coolant affected the inflammation process by decreasing the neutrophil number in rat gingiva.

*E. hirta* contains secondary metabolites, such as flavonoids, tannins, terpenoids, and saponins, which can have anti-inflammatory effects. The flavonoids in *E. hirta* influence the mean number of neutrophils through several mechanisms, including the inhibition of cyclooxygenase, which enables prostaglandin synthesis, and lipoxygenase, thus promoting the synthesis of beta-4 leukotrienes, neutrophil degranulation (exocytosis), and induction of apoptosis from neutrophils.^26^, ^27^, ^28^, ^29^ The inhibition of prostaglandin synthesis and leukotriene-4 in neutrophil chemoactivity induces proinflammatory cytokines, damages collagen fibers, and causes bone destruction, thereby decreasing the average number of neutrophils.^27^^,^^30^ The mechanism of inhibition of the release of neutrophil granules can decrease neutrophil numbers, because neutrophil granules contain pro-inflammatory toxic mediators.^31^ Moreover, neutrophil apoptosis accelerates the healing process of gingivitis through the secretion of mediators preventing neutrophil recruitment, including annexin 1. Annexin 1 induces the clearance of neutrophils that have undergone apoptosis by proinflammatory macrophages and efferocytosis. This efferocytosis mechanism induces the cessation of cytokine production and proinflammatory mediators, and converts the macrophage phenotype to macrophage type 2.^32^ Flavonoids can also form complexes with microbial proteins, thereby inhibiting enzymes involved in cell replication.^33^ The bioactivities of *E. hirta* metabolites may indicate the potential mechanism underlying the decrease in neutrophil count.

The decrease in neutrophil number in the treatment groups was also indirectly influenced by the biological effects of tannin, an antibacterial agent. Cell membranes of gram-positive bacteria are more sensitive to active substances in the extract than gram-negative bacteria, because the outer membrane of gram-positive bacteria is composed of thick, porous peptidoglycans that facilitate the absorption and penetration of the extract. In contrast, absorption and penetration of extracts in gram-negative bacteria is hindered by the bacterial cell membrane bilayer, comprising a thin layer of peptidoglycan and an outer layer composed of lipopolysaccharides, lipoproteins, and phospholipids.^33^^,^^34^ In addition, tannin has antibacterial properties, through inhibition of reverse transcriptase and DNA topoisomerase enzymes, thus preventing bacterial cell wall synthesis and bacterial growth. Tannins also act as antioxidants that bind free oxygen species and reactive radicals; consequently, they tend to be stable and to have minimal adverse effects. Tannins also accelerate wound contraction, capillary growth, and fibroblast proliferation.^35^

Saponins function as antibacterial agents (through the initiation of hemolysis of bacterial cells) and stimulate angiogenesis by upregulating the expression of vascular endothelial growth factor (VEGF), thus inducing endothelial cell migration.^34^, ^35^, ^36^, ^37^ Furthermore, terpenoids have anti-inflammatory activity through molecular targets such as pro-inflammatory cytokines, transcription factors, the autophagy machinery, reactive oxygen species, membrane receptors, and other inflammatory mediators. Terpenes act as anti-inflammatory agents through various cell signaling pathways, thereby increasing effectivity of the anti-inflammatory activities.^38^

Angiogenesis is the process through which new blood vessels form from old blood vessels. Angiogenesis provides an adequate supply of oxygen and nutrients as well as inflammatory cells in regenerating tissue; eliminates necrotic tissue; and increases vascular permeability, endothelial proliferation, and macrophage chemotaxis. The formation of new blood vessels is characterized by the release of protease enzymes from endothelial cells, which are activated by angiogenic factors.^37^

The average number of blood vessels on day 3 was lowest, owing to the migration of endothelial progenitor cells to the blood circulation and granulation tissue. Formation of new blood vessels occurs through the degradation of the extracellular matrix, migration, proliferation of endothelial cells, and synthesis of new extracellular matrix. This stage is followed by controlled blood vessel maturation to meet tissue needs. After adequate tissue formation, migration and proliferation of endothelial cells decreases, and apoptosis removes excess endothelial cells.^36^^,^^37^

The average angiogenesis increased on days 5 and 7, and was highest on day 14 in all groups, but tended to be higher in the treatment than the control groups, presumably because flavonoids and saponins in *E. hirta* accelerated angiogenesis through VEGF stimulation. Angiogenesis, an important process in healing gingivitis, involves growth factors, particularly platelet-derived growth factor (PDGF), VEGF, and bFGF, as well as endothelial progenitor cells. Angiogenesis begins with the degradation of old blood vessels that provide progenitor cells. The progenitor cells in turn migrate distally to the capillaries and elicit angiogenic stimulation. This migration of progenitor cells is influenced by VEGF, which is produced by macrophages when blood vessels are damaged and hypoxic. VEGF expression is also regulated by growth factors such as transforming growth factor-β (TGF-b) and PDGF. The proliferation of endothelial cells begins with lumen formation through intracellular and intercellular mechanisms, including recruitment and proliferation of pericytes and smooth muscle cells to support the endothelial wall and perform additional functions.^36^, ^37^, ^38^, ^39^

Increased angiogenesis plays an important role in accelerating tissue healing, by increasing the supply of oxygen, nutrients, and inflammatory cells to injured tissues. Increased angiogenesis is believed to prevent chronic hypoxia, eliminate tissue-damaging microorganisms, form granulation tissue, and increase re-epithelialization to cover the injured area. These processes accelerate the inflammatory and proliferative phases and entry into the remodeling phase, thus restoring gingival integrity.^37^

Furthermore, the use of chitosan as an encapsulant affects the mean neutrophil count. Chitosan inhibits bacterial cell growth and damages bacterial cells, particularly gram-positive bacteria, such as *Porphyromonas gingivalis*.^18^ Interactions between chitosan and bacterial cells are influenced by the positive charge of chitosan and the negative charge of the bacterial cell membrane. These interactions cause opening of tight junction epithelium in bacterial cell membranes, thus resulting in loss of intracellular components from bacteria.^19^ The size of the *E. hirta* extract coolant particles encapsulated in nanometer-sized chitosan influences adhesion to, and penetration of, cell membranes: a larger nanoparticle surface facilitates interaction with larger amounts of cells and biomolecules.^25^ Decreases in neutrophil counts also occurred in the positive control group treated with 2% iodine glycerin coolant, an effective antibacterial and antifungal agent.^39^, ^40^, ^41^, ^42^

Scaling and root planning have been found to significantly decrease and debride periodontopathogen accumulation in the periodontal pocket, and have been the gold standard non-surgical periodontal therapy, including gingivitis treatment. The high-frequency vibration of ultrasonic scaling effectively removes calculus deposits, thus significantly decreasing damage-inducing microorganisms.^43^ Ultrasonic scaling is used to treat calculus deposits, because it does not damage the cementum, produces the least surface roughness, and is rapid.^44^ Mechanical removal of periodontopathogens might be insufficient to achieve homeostasis to prevent tissue damage; thus, a coolant with antibacterial and anti-inflammatory activity might facilitate therapeutic effects by reaching deep gingival tissue and inducing healing.^45^

A combination of mechanical removal with ultrasonic scaling and chemical removal with bioactive agents has been used to achieve better gingivitis healing. A previous study has used chlorhexidine, povidone iodine, and cinnamon extract as an ultrasonic coolant and has reported a decreased bacterial count.^46^^,^^47^ Beyond the potential anti-bacterial activities of those coolants in gingivitis treatment, little research has focused on their potential in treating gingival inflammation. This study reported the potential development of *E. hirta* as an ultrascaler coolant with anti-inflammatory and tissue-healing inducing properties, marked by a decreased neutrophil count and promotion of angiogenesis. This potential should be further explored by using various markers to thoroughly understand the anti-inflammatory mechanisms of this ultrasonic scaler coolant.

## Conclusion

Our study revealed that use of *E. hirta* nanoextract as a piezoelectric ultrasonic coolant achieved good therapeutic results in promoting healing of gingivitis, particularly at a 25% concentration. *E. hirta* nanoextract effectively resolved inflammation in gingivitis, particularly affecting neutrophils and angiogenesis. Our findings confirmed that *E. hirta* nanoextract may potentially be used as in gingivitis treatment. Further studies should be conducted to isolate each component of *E. hirta* and assess its pharmacodynamic and pharmacokinetic properties to advance applications in the treatment of gingivitis.

## Source of funding

This study was funded by Dana Masyarakat Research grant 2019 number 4326/UN1/FKG1/Set.KG1/PT/2019, Faculty of Dentistry, 10.13039/501100012521Universitas Gadjah Mada, Republic of Indonesia.

## Conflicts of interest

The authors have no conflict of interest to declare.

## Ethical approval

This research was conducted in accordance with the Guide for the Care and Use of Laboratory Animals, National Health Research and Development Ethics Standard and Guidelines Councils (207), Minister of Health, Republic of Indonesia. The protocol was approved and received ethical clearance from the ethics committee of the Faculty of Medicine, Universitas Gadjah Mada, Republic of Indonesia, under number KE/FK/0827/EC/2019 (approval date July 19, 2019).

## Authors contributions

AN designed the study, acquired funding, and revised the draft article. MDW conducted research; organized, analyzed, interpreted the data; and wrote the initial draft of the article. LU and RNSQA conducted research and co-wrote the initial draft of the article. AS and MHS revised the draft. All authors have critically reviewed and approved the final draft and are responsible for the content and similarity index of the manuscript.

## References

[1] Newman M.G., Takei H.H., Klokkevold P.R., Carranza F.A. (2015).

[2] Kartiyani I., Santoso O. (2010). The effect of exposure to sulfur vapor on the incidence of gingivitis study of sulfur mine workers at Mount Welirang, Pasuruan, East Java. J PDGI.

[3] Reddy S. (2017).

[4] Nurma Azuro H., Yunus M., Warih Gayatri R. (2021). Early detection of periodontitis in public health Centres in Malang: an overview. KnE Life Sci.

[5] Marry Miller, Crispian Scully (2015).

[6] Ud-Din S., Bayat A. (2016 Aug). Non-invasive objective devices for monitoring the inflammatory, proliferative and remodelling phases of cutaneous wound healing and skin scarring. Exp Dermatol.

[7] Chatterje A., Baiju C.S., Bose S., Shetty S.S. (May 2013). Clinical uses and benefits of ultrasonic scalers as compared to Curets: a review. J Oral Health Community Dent.

[8] Peeran S.W., Ramalingan K. (2021).

[9] Mirossay L., Varinská L., Mojžiš J. (December 2017). Antiangiogenic effect of flavonoids and chalcones: an update. Int J Mol Sci.

[10] Ghosh P., Ghosh C., Das S., Das C., Mandal S., Chatterjee S. (2019). Botanical description, phytochemical constituents and pharmacological properties of *Euphorbia hirta* Linn: a review. IJHSR.

[11] Nyeem M.A.B., Haque M.S., Akramuzzaman M., Siddika R., Sultana S., Islam B.M.R. (2017). Euphorbia hirta Linn. A wonderful miracle plant of mediterranean region: a review. J Med Plants Stud.

[12] Nurbaiti, Fajar A., Hikmah (2018). The effectiveness of Patikan Kebo (*Euphorbia hirta* L.) leaf extract compared with 10% povidone iodine against epithelialization thickness in incision wounds in male white rats. Tunas Med J Kedokt Kesehat.

[13] Nofikasari I., Rufaida A., Aqmarina C.D., Failasofia F., Fauzia A.R., Handajani J. (2017). Effect of topical application of fragrant pandan extract gel on gingival wound healing. Maj Kedokt Gigi Indones.

[14] Ahmad S., Sultan P., Ashour A.E., Khan T.H., Attia S.M., Bakheet S.A. (2013 Oct). Modulation of Th1 cytokines and inflammatory mediators by Euphorbia hirta in animal model of adjuvant-induced arthritis. Inflammopharmacology.

[15] Martien R., Adhyatmika, Irianto I.D.K., Farida V., Sari D.P. (2012). Technology developments nanoparticles as drug. Maj Farm.

[16] Kargozer S., Baiono F., Hamzehlou S., Hamblin M.R., Mozafari M. (2020 Jul 21). Nanotechnology for angiogenesis: opportunities and challenges. J Chem Soc Rev.

[17] BPOM RI (2013).

[18] Arancibia R., Maturana C., Silva D., Tobar N., Tapia C., Salazar J.C. (2013 Aug). Effects of chitosan particles in periodontal pathogens and gingival fibroblasts. J Dent Res.

[19] Mohammed M.A., Syeda J.T.M., Wasan K.M., Wasan E.K. (2017 Nov). An overview of chitosan nanoparticles and its application in non-parenteral drug delivery. Pharmaceutics.

[20] Arifin W.N., Zahiruddin W.M. (2017). Sample size caculation in animal studies using resource equation approach. Malays J Med Sci.

[21] Prasetyorini, Hasan A.E.Z., Rofiqah S. (2011). Penerapan teknologi nanopartikel propolis trigona Spp asal Bogor sebagai antibakteri *Escherichia coli* secara in-vitro. Ekologia.

[22] Balakumar K., Raghavan C.V., Selvan N.T., Prasad R.H., Abdu S. (2013 Dec). Self nanoemulsifying drug delivery system (SNEDDS) of rosuvastatin calcium: design, formulation, bioavailability and pharmacokinetic evaluation. Colloids Surf B Biointerfaces.

[23] Nugroho A.M., Elfiah U., Normasari R. (2016). Effect of extract gel and powder of cucumber (*Cucumis sativus*) on angiogenesis in healing second degree burns in Wistar rats. Pustaka Kesehat.

[24] Raziyeva K., Kim Y., Zharkinbekov Z., Kassymbek K., Jimi S., Saparov A. (2021). Immunology of acute and chronic wound healing. Biomolecules.

[25] Pesce M., Patruno A., Speranza L., Reale M. (2013 Mar). Extremely low frequency electromagnetic field and wound healing: implication of cytokines as biological mediators. Eur Cytokine Netw.

[26] Neck J. van, Tuk B., Barritault D., Tong M., Davies J. (2012). Tissue regeneration – from basic Biology to clinical application.

[27] Lucas C.D., Allen K.C., Dorward D.A., Hoodless L.J., Melrose L.A., Marwick J.A. (2013 Mar). Flavones induce neutrophil apoptosis by down-regulation of Mcl-1 via a proteasomal-dependent pathway. FASEB J.

[28] Moro M.G., Oliveira M.D.D.S., Oliveira L.R. de, Teixeira S.A., Muscará M.N., Spolidorio L.C. (2019). Effects of selective versus non-selective COX-2 inhibition on experimental periodontitis. Braz Dent J.

[29] Riansyah Y., Mulqie L., Choesrina R. (2019). Anti-inflammatory activity test of ethanol extract of purple sweet potato leaves (*Ipomoea batatas (L.) Lamk*) against male Wistar rats. Pros Penelit SPeSIA.

[30] Sankari S.L., Babu N.A., Rani V., Priyadharsini C., Masthan K.M.K. (2014 Jul). Flavonoids – clinical effects and applications in dentistry: a review. J Pharm BioAllied Sci.

[31] Lopes D.E.M., Jabr C.L., Dejani N.N., Saraiva A.C., de Aquino S.G., Medeiros A.I., Rossa Junior C. (2017 Dec 5). Inhibition of 5-lipoxygenase attenuates inflammation and BONE resorption in lipopolysaccharide-induced periodontal disease. J Periodontol.

[32] Sheshachalam A., Srivastava N., Mitchell T., Lacy P., Eitzen G. (2014 Sep 19). Granule protein processing and regulated secretion in neutrophils. Front Immunol.

[33] Farhadi F.F., Bahman K., Mehrdad I., Milad I. (2018 October). Antibacterial activity of flavonoids and their structure-activity relationship: an update review. Phytother Res.

[34] Lingga A.R., Pato U., Rossi E. (2016). Antibacterial test of kecombrang (*Nicolaia speciosa horan*) stem extract against *Staphylococcus aureus* and *Escherichia coli*. JOM Faperta.

[35] Shita A.D.P., Dharmayanti A.W.S., Meilawaty Z., Lestari M., Mazaya I.M.A. (2023). Increasing fibroblast and gingival collagen density in periodontitis rats by using cassava leaf extract. J Taibah Univ Med Sci.

[36] Dorr T., Moynihan P.J., Mayer C. (2019). Editorial: bacterial cell wall structure and dynamics. Front Microbiol.

[37] Nofikasari I., Rufaida A., Aqmarina C.D., Failasofia Fauzia, Handajani dkk (2016). Effect of topical application of pandan Wangi extract gel on gingival wound healing. Maj Kedokt Gigi Indones.

[38] Prado-Audelo M.L.D., Cortes H., Caballero-Floran I.H., Gonzales-Torres M., Escutia-Guadarrama L., Bernal-Chavez S.A., Giraldo-Gomez D., Magana J.J., Gerardo Leyva-Gomez (2021). Therapeutic applications of terpenes on inflammatory diseases. Front Pharmacol.

[39] Ortega-Gómez A., Perretti M., Soehnlein O. (2013 May). Resolution of inflammation: an integrated view. EMBO Mol Med.

[40] Cekici A., Kantarci A., Hasturk H., Van Dyke T.E. (2014 Feb). Inflammatory and immune pathways in the pathogenesis of periodontal disease. Periodontol 2000.

[41] Chen Y., Tian L., Yang F., Tong W., Jia R., Zou Y., Yin L., Li L., He C., Liang X., Ye G., Lv C., Song X., Yin Z. (2019 Jul 1). Tannic acid accelerates cutaneous wound healing in rats via activation of the *ERK 1/2* signaling pathways. Adv Wound Care.

[42] Xiumin L., Dey G., Bin L., Cuifei Y., Fengyuan L. (2006). Comparison of iodophor and iodine glycerin in the treatment of experimental rat gingivitis. J Cap Med Univ.

[43] Oza R., Sharma V., Multani P., Balsara K., Bajaj P., Dhadse P. (2022). Comparing the effectiveness of ultrasonic instruments over manual instruments for scaling and root planing in patients with chronic periodontitis: a systematic review and meta-analysis. Cureus.

[44] Yan Y., Zhan Y., Wang X., Hou J. (2020). Clinical evaluation of ultrasonic subgingival debridement versus ultrasonic subgingival scaling combined with manual root planing in the treatment of periodontitis: study protocol for a randomized controlled trial. Trials.

[45] Gupta I., Tripathi A., Gupta R., Ranjan P., Gupta S., Das N. (2022). Clinical evaluation of 10% azadirachta indica mouth rinse as a subgingival irrigant along with ultrasonic scaling for the treatment of chronic gingivitis and chronic periodontitis. Int J Health Sci.

[46] Mamajiwala A., Sethi K., Mahale S., Raut C., Karde P. (2019). Comparative evaluation of the chlorhexidine and cinnamon extract as ultrasonic coolant for reduction of bacterial load in dental aerosols. J Indian Soc Periodontol.

[47] Jawade R. (2016). Comparative evaluation of two different ultrasonic liquid coolants on dental aerosols. J Clin Diagn Res.

