# Supplementary material for: Euphorbia hirta nanoextract as a piezoelectric ultrasonic scaler coolant in gingivitis treatment in a Wistar rat model
Source: J Taibah Univ Med Sci. 2023 Oct 6;19(1):1–9. doi: 10.1016/j.jtumed.2023.09.004 (PMC10585296; doi:10.1016/j.jtumed.2023.09.004)
Supplement: Multimedia component 1 [file mmc1.docx]

| **Supplementary 1. Kolmogorov-Smirnov normality test results** | | | | | | | |
| --- | --- | --- | --- | --- | --- | --- | --- |
| **Group** | **Neutrophils** | | | | **Angiogenesis** | | |
|  | **df** | **Statistic** | | **Sig.** | | **Statistic** | **Sig.** |
| Negative control | 15 | 0.891 | 0.183* | | | 0.903 | 0.200* |
| 25% | 15 | 0.914 | 0.200* | | | 0.928 | 0.200* |
| 30% | 15 | 0.919 | 0.200* | | | 0.940 | 0.183* |
| 35% | 15 | 0.898 | 0.110* | | | 0.912 | 0.165* |
| Positive control | 15 | 0.844 | 0.263* | | | 0.895 | 0.200* |
| (*): significance (p>0.05) | | | | | | | |

| **Supplementary 2.** Shapiro-Wilk homogeneity test results | | | | | |
| --- | --- | --- | --- | --- | --- |
|  | **Levene’s statistic** | **df1** | **df2** | **Sig.** | |
| Neutrophils | 1.836 | 4 | 70 | 0.130* | |
| Angiogenesis | 2.391 | 4 | 70 | 0.776* | |
| (*): significance (p>0.05) | | | | |  |
